# Management of Tongue-Tie Using Diode Laser for Speech Clarity: A Case Report

**DOI:** 10.7759/cureus.46667

**Published:** 2023-10-08

**Authors:** Sneha Dare, Unnati Shirbhate, Pavan Bajaj

**Affiliations:** 1 Department of Periodontics, Sharad Pawar Dental College, Datta Meghe Institute of Higher Education and Research, Wardha, IND

**Keywords:** lingual frenum, laser, frenectomy, tongue-tie, ankyloglossia

## Abstract

A congenital condition called ankyloglossia, or tongue tie, is characterized by an excessively short or tight lingual frenum that restricts the tongue’s movement and flexibility. Although ankyloglossia, or tongue tie, is not a serious sign, it can cause a variety of challenges, such as difficulty with newborn feeding, speech problems, and many mechanical and social problems since there are restricted tongue movements, such as protrusion of the tongue. It is recommended to get a lingual frenectomy to treat ankyloglossia. A 24-year-old female patient reported to the Department of Periodontics with class II, moderate lingual tie, or ankyloglossia. Under local anesthesia, the lingual frenectomy is performed with a diode laser by placing a hemostat across the frenal attachment at the base of the tongue, and an incision is made. The laser surgery took less time and was more comfortable for the patient because there was less discomfort. There was no postoperative pain or hemorrhage. The above case report can appreciate the normal frenal attachment that is more than 16 mm, and the patient can hold the tip of the tongue and function comfortably. A follow-up visit after three months revealed normal frenal attachment and complete healing of the frenum. This case report demonstrates unequivocally that lingual frenectomy using a diode laser has advantages over traditional procedures in that it reduces or eliminates postoperative pain and minimizes hemorrhage and swelling.

## Introduction

The lingual frenum is a thin strip of tissue that runs vertically from the mouth's floor to the tongue's undersurface. A "V"-shaped mass of tissue in the floor of the mouth surrounds the base of the frenum and houses several salivary gland ducts. The second-largest ducts, Wharton's ducts, are situated in the middle, directly in front of the lingual frenum attachment. Salivary glands in the submandibular and sublingual regions are drained. Varicosities, or superficial veins, pass across the frenum's base. It is usual for them to be there, and as the patient gets older, they become more noticeable [1]. Ankyloglossia, often known as tongue tie, is an abnormal condition that affects the lingual frenum. The frenum may be retained tight to the gingival borders of the lower anterior teeth and attached at or near the tip of the tongue. Gingival tissue recession has been correlated with high muscle attachment and frenal pull. Rarely, it crosses the mandibular alveolus and spreads across the mouth’s floor. Usually, there is no diastema between the mandibular central incisors and the lingual frenum [2-5].

Using Kotlow's assessment, the four categories of ankyloglossia are the following: Class I: mild ankyloglossia between 12 and 16 mm; Class II: moderate ankyloglossia between 8 and 11 mm; Class III: severe ankyloglossia measuring between 3 and 7 mm; Class IV: complete ankyloglossia measuring under 3 mm. A simple anatomical classification based on measuring the “free tongue” length between the frenulum attachment to the tongue and the tongue’s tip is considered for measurement [6].

A diastema between the mandibular central incisors or the tissue behind them may tear away due to ankyloglossia, which can produce tension in the floor of the mouth. Researchers in periodontics and maxillofacial surgery have suggested many methods for managing patients with ankyloglossia. Bipolar diathermy, lasers, and surgical blades are some of the ways [2-6]. Traditional surgical frenectomies have drawbacks even when they yield positive results. Surgery performed on the ventral portion of the tongue may harm the lingual nerve and result in numbness near the tongue's tip. Sometimes, suturing on the ventral surface of the tongue might block Wharton's duct, resulting in swollen mandibles. Additionally, the "wicking effect" of contamination from sutures might result in a subsequent infection. The multifilament sutures produce more capillary action, or "wicking effect," than the monofilaments, which draw more bacteria into the tissues and induce a stronger inflammatory response. This necessitates the recommendation of postoperative antibiotics [6].

Due to its monochromatic, coherent, and collimated nature, laser light precisely directs an energy burst to the target place. Histological analysis has shown that LASER wounds contain substantially fewer fibroblasts. Faster healing is the result of less scarring and wound contraction. Due to the stimulation of clotting factor VII production and the closing of capillaries by denaturing proteins, LASER wounds bleed very little or not at all. Compared to the knife procedure, LASER frenectomy offers a better postoperative impression of discomfort and function [6,7].

## Case presentation

**Figure 1:**
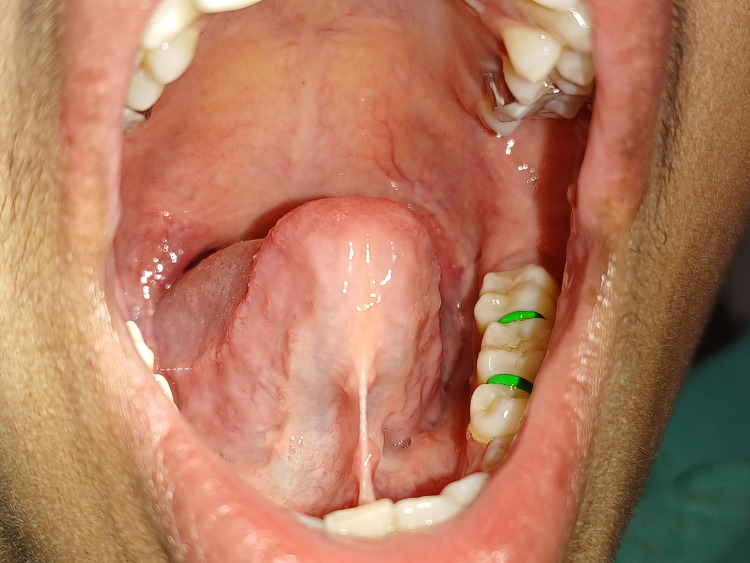
Pre-operative view showing restricted tongue movement

A 24-year-old female patient complained of speech difficulties and difficulty fully protruding her tongue when she visited the Department of Periodontics. The patient could not pronounce words such as “s, n, t, d, j, zh, ch, th” and could not touch the tip of the tongue until the incisive papilla. On clinical inspection, ankyloglossia was noticed. Figure 1 depicts the pre-operative view of a tongue tie with restricted tongue movement. 

**Figure 2:**
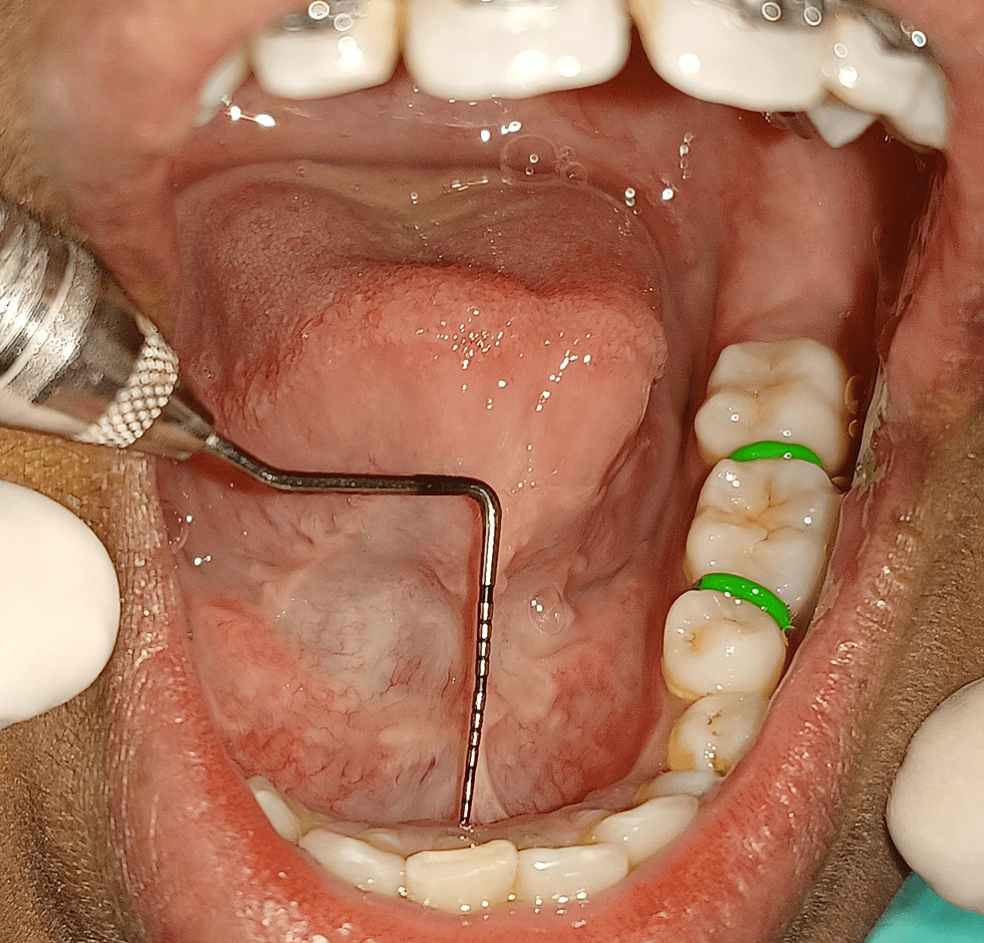
Measurement showing class II ankyloglossia that is 8 mm

Class II: 8 mm of lingual frenum attachment, or mild ankyloglossia, was present on the measurement (Figure 2). 

**Figure 3:**
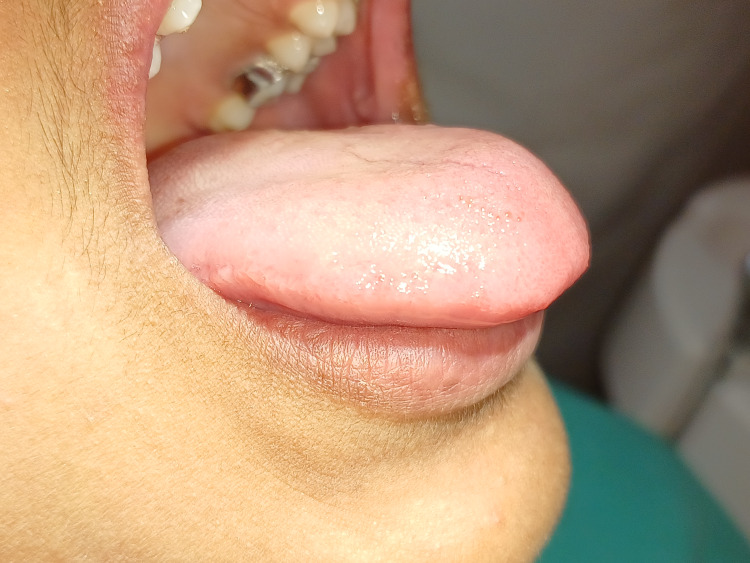
Pre-operative view showing restricted tongue protrusion

The patient also had difficulty with tongue protrusion (Figure 3). 

**Figure 4:**
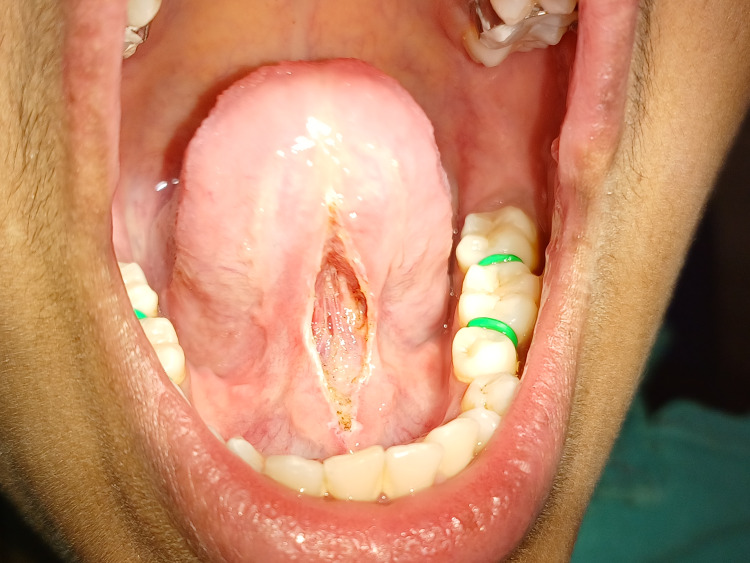
Lingual frenectomy performed with a diode laser

Diode laser lingual frenectomy was intended for the same. The manufacturer's recommended laser safety protocol is strictly followed. The patient, assistant, and operator wore safety eyewear during surgery. The lingual frenectomy is performed with a diode laser of wavelength 880 nm and 0.8 W power under local anesthesia infiltration solution of 2% lignocaine with 1:200,000 adrenaline by placing a hemostat over the frenal attachment at the base of the tongue and making an incision (Figure 4).

**Figure 5:**
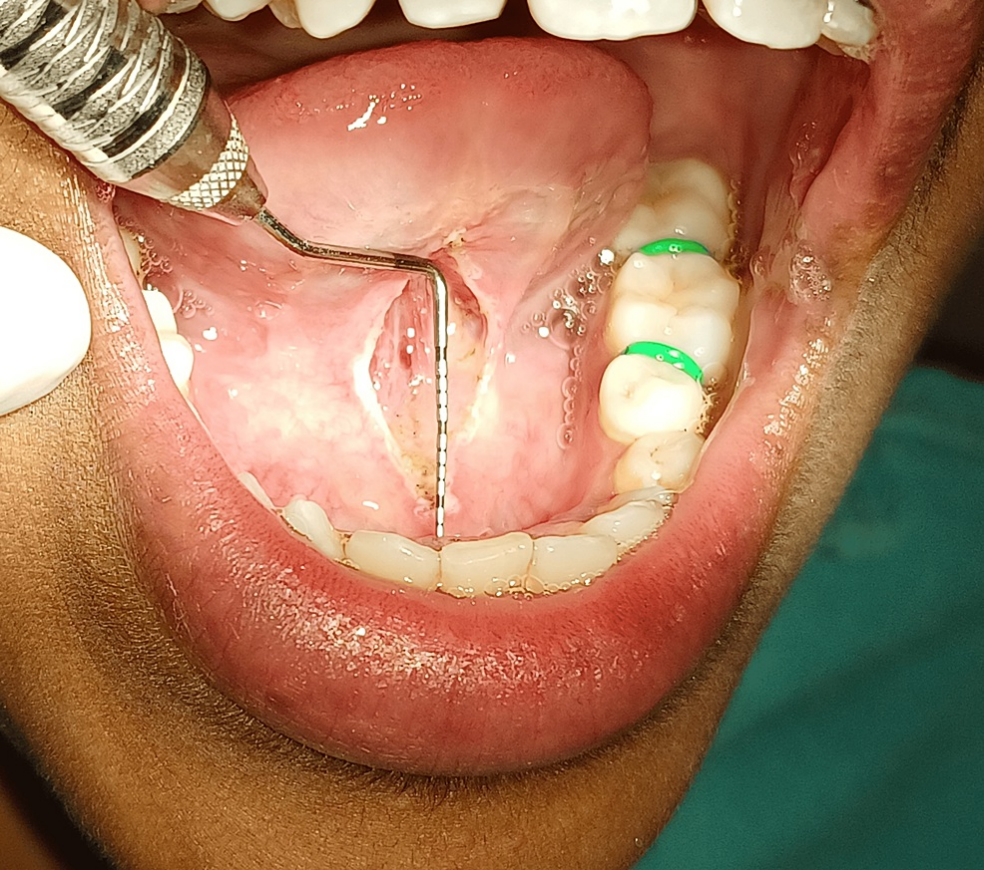
Post-operative view showing measurements at the operating site

The frenum was released by moving the laser's tip in a brushing motion from its apex to its base. The debris on the surface of the ablated tissue was continuously removed by wiping it with a piece of wet gauze. By doing this, the burned tissue is treated, and the surrounding soft tissue is shielded from further thermal harm. The frenum's connection to the alveolar ridge was also removed to avoid pulling on the gingiva. No bleeding or discomfort was observed during the surgical procedure. No sutures were placed. Post-operative measurements at the operating site within the normal range can be seen in Figure 5. 

**Figure 6:**
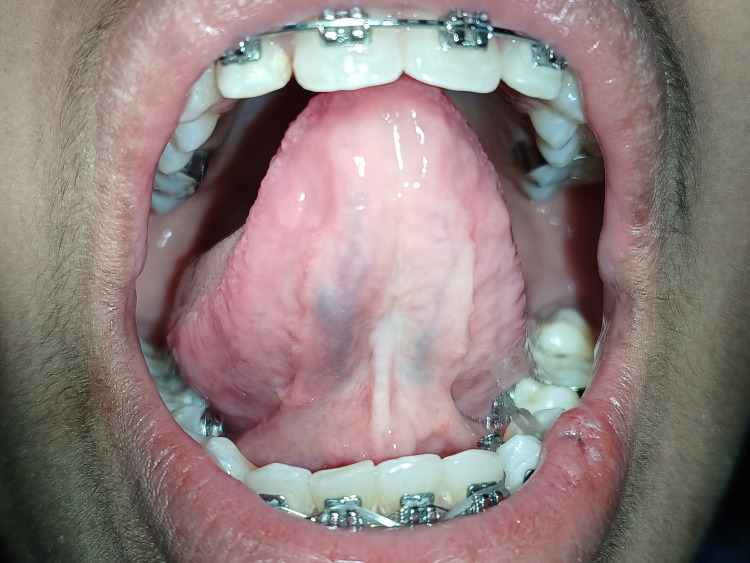
Follow-up recall examination after three months reveals complete satisfactory wound healing and no restricted tongue movement

**Figure 7:**
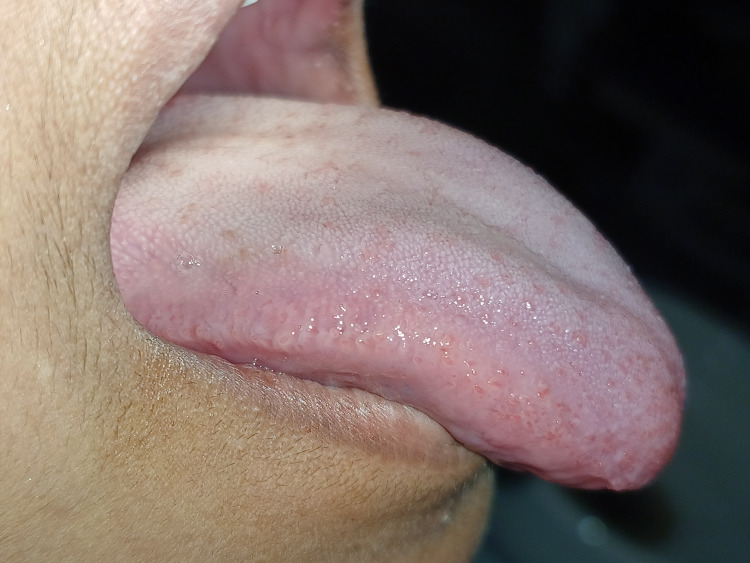
A follow-up examination after three months reveals no restricted tongue protrusion

After a one-week follow-up, the patient was rechecked, and the recovery was satisfactory. The patient was educated and advised to do orofacial musculature and tongue exercises after 2 -3 weeks postoperatively. The patient was recalled after 1 and 3 months, respectively, which reveals satisfactory wound healing and no restricted tongue movements. Also, the patient could touch her tongue until the incisive papillae protruded her tongue and pronounced words such as “s, n, t, d, j, zh, ch, and th” (Figures 6, 7) on recall examination.

**Figure 8:**
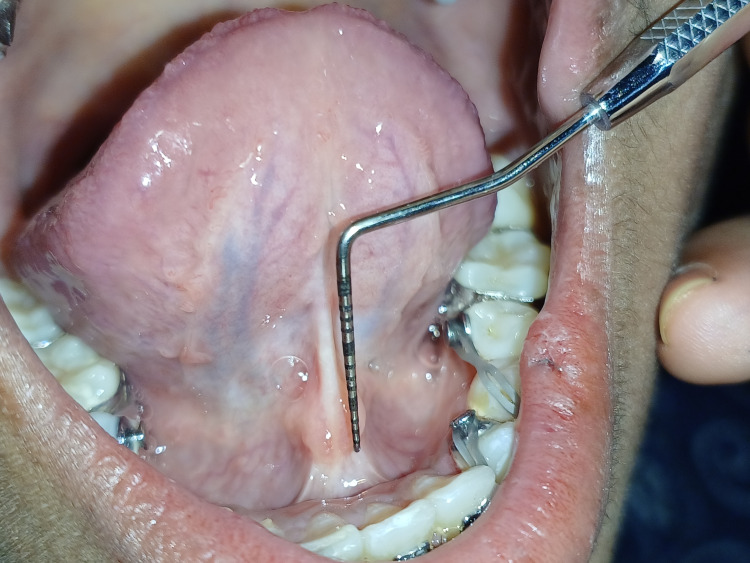
Recall examination after three months showed more than 16 mm of measurement at the operating site

Measurements were taken at a three-month follow-up of the operated site, which resulted in more than 16 mm of frenal attachment (Figure 8). 

In this case presentation, the patient had some speech and tongue-movement-related problems before surgery and was pleased with the remarkable outcomes after the surgery. The patient had no history of postoperative bleeding due to laser therapy, and no such discomfort presented, which gave confidence to the patient and made this case expressive for speech clarity. 

## Discussion

A frenum is a muscle or tissue fold connecting the tongue, cheeks, and lips to the jawbone. Other names for it include frenulum, frenula, and frena. A congenital condition called ankyloglossia, or tongue tie, is characterized by an excessively short or tight lingual frenulum that limits the tongue's ability to move [7,8]. The prevalence ranges between 4.2% and 10.7% [5]. The surgical blade, bipolar diathermy, and laser are only a few recommended therapy techniques mentioned in the literature. To avoid tongue deformities and speech issues, lingual frenum interventions should be carried out as soon as possible if a tongue deformity is present. To provide effective postoperative care and obtain favorable clinical results and overall patient satisfaction, it is necessary to have comprehensive knowledge and an understanding of the varied causes of complications associated with lingual frenectomy [7]. There are so many drawbacks to surgical frenectomy procedures, which include bleeding, infection at the site, injury to salivary ducts near the tongue, pain, swelling, and scar tissue formation [6,7]. Diode laser surgery has been identified as the optimal therapeutic approach for ankyloglossia across various age groups due to its safety, noninvasive nature, conclusive outcomes, and minimal complications. Furthermore, surgical treatments with a diode laser are less technique-sensitive than traditional scalpel surgery [8]. The diode laser's unique characteristic allows for the precise removal of a thin epithelium layer and the subsequent induction of a sterile inflammatory response. It also causes minimal harm to the periosteum and bone beneath the gingiva being treated [9-11]. Laser frenectomy could be considered a viable alternative to traditional surgical methods, particularly in patients with difficulties that preclude them from enduring longer-term procedures and patients susceptible to pain [12].

## Conclusions

To avoid tongue deformities and speech issues, lingual frenum interventions should be carried out as soon as possible if a tongue deformity is present. This case report demonstrates unequivocally that lingual frenectomy using a diode laser has advantages over traditional procedures in that it reduces or eliminates postoperative pain and minimizes hemorrhage and swelling.
